# Zoning a Great City

**Published:** 1924-12

**Authors:** 


### ZONING A GREAT CITY.

### TOWN-PLANNING AT SHEFFIELD.

In addition to his Reports on Regional Planning at Doncaster and at Deeside, Professor Patrick Abercrombie, of Liverpool University, has now issued, through Messrs. Hodder and Stoughton and the University Press of Liverpool, " A Civic Survey of Sheffield," with suggestions towards a Development Plan, prepared with Mr. R. H. Mattocks. Like its predecessors, this is an admirably written and well-illustrated volume, which has been drawn up with the assistance of many prominent Sheffield men, including the late Medical Officer of Health, Dr. Scurfield and his successor, Dr. Wynne. Sheffield is described as perhaps " the largest purely manufacturing town in the country." Its simple aim indeed is to be a successful manufacturing community. " There can be no doubt as to its main occupation, as there is for example at Chester, where county town, market centre, cathedral city, manufacturing borough, residential and recreational resort are all of importance, and it is almost impossible to say which is fundamental." At Sheffield the needs of the worker are paramount.

### Natural Selection.

Hitherto the town has grown up at its own sweet will :—

All the larger aspects of the town's existence have been left to take care of themselves, such as the placing of the heavy and smoky works on the extreme east and the choice for the larger houses of the attractive country on the west ; and no one can dispute that these natural selections are in the main sound. If any artificial direction is to be introduced into this stage of the city's existence, it must seek to make use of them, heightening indeed their character and removing obstructions to their complete realisation, unless, of course, it is found that some major requirement for the common good is infringed.

### Health and Housing.

With regard to health and housing conditions, " density is clearly the factor most consistently causative of high mortality." The proximity of smoky works to dwelling houses is also exceedingly unfavourable to health. The number of back-to-back houses still existing in Sheffield is 16,529, but this type of house has not been erected since 1864 :—

These back-to-back houses were not generally built facing on to closely spaced parallel streets as it is found in other towns where they were erected more recently ; the usual arrangement is for one set of houses to face on to the roads and the other on to an internal court, entered through an archway. In many cases one side of the court is occupied by a factory and small workshops are placed in it.

### Similar Interests.

The function of this zoning plan is to preserve a just balance between the industrial and business interests of Sheffield on the one hand, and the residential on the other :—

There is no antagonism between the two as the same people are concerned in each, but it is sectional action in an unconsidered way that does the damage. There is, for example, the danger of preventing industrial expansion by using the limited amount of land suitable for certain trades, for houses (from which Sheffield is suffering in the Don Valley to-day) ; there is, again, the danger of single factories finding a suitable site in one of the suburbs owing to the greater use of motor transport, and thereby damaging a large neighbourhood.

### Improving Central Sheffield.

A large area has been set aside for industrial development, and certain shopping and office streets are protected from light trade factories being set up in them for " there can be no monumental or dignified core to the city if factories, however well run, can be placed anywhere." Suggested improvements for Central Sheffield include bringing the two railway lines —the Midland and Great Central—into a combined terminus in the centre of the city, with a spacious Station Place approached over Exchange Street by a bridge. New and better markets are required ; the increase of central shopping streets is a pressing problem.

### Satellite Suburbs.

Sheffield is surrounded by much beautiful country, and Professor Abercrombie is of opinion that it is wiser to incorporate in the Park system these many belts of delightful woodlands than to create new parks. " There are not many towns of the size of Sheffield which can to-day plan a footpath to lead from the centre to the open moors." It is this very accessibility to the country that has perhaps prevented the evolution of satellite suburbs :—

To a student investigating Sheffield it must always appear a matter of surprise that this satellite suburbanising has not occurred more spontaneously, and particularly when one discovers the astonishing beauty within half-an-hour's railway journey of the centre. Two deterrent causes then appear. The bad placing of the two main railway stations and the wind-swept viaduct of Victoria and the down-in-a-hole remoteness of the Midland . . . are sufficient to put off any business man or his wife ; the other cause is the accessibility of charming suburbs suitable for all sizes of houses within the city boundary. There has not so far been much need to go far afield. Something, indeed, can be done within the present boundary to encourage neighbourhood centres at naturally suitable points. But a really satisfactory solution of the future suburban question of Sheffield can only be undertaken on the basis of a much wider regional control.

There are many sections in the Report on which we have been unable to touch, but we commend its study to all who are desirous of seeing an improvement in the conditions of those who live in the great industrial towns of England.

---

### OLDEST LYING-IN HOSPITAL.

At a meeting of the Royal Society of Medicine recently, Dr. G. C. Peachey read a paper on " The Provision for Lying-in Women in London up to the Middle of the Eighteenth Century," in which he stated that he had established the fact that Queen Charlotte's Maternity Hospital was the earliest lying-in hospital in the British Isles. Amongst the Sloane MSS. in the British Museum he had found a letter dated May 17, 1739, from Sir Richard Manningham, the foremost obstetrician of his time, from which it appeared that in the summer of 1739 he opened a house in Jermyn Street next to his own residence for the reception of twenty-five lying-in women. It was supported by voluntary contributions. Dr. Peachey traces the hospital at different addresses until its removal in 1813 to its present site in the Marylebone Road. In 1809, when the hospital was in very low water, the Duke of Sussex became president for life and the institution was reorganised and re-established under his direction. In the following year Queen Charlotte became patron, and the name of the hospital was changed from the General Lying-in Hospital to the Queen's Lying-in Hospital, and subsequently to Queen Charlotte's Lying-in Hospital. An amended Royal Charter has changed the name to Queen Charlotte's Maternity Hospital.

